# ‘Foreseeing well-being’: developing a physical health strategic vision across a large mental health trust ‘foreseeing well-being’: developing a physical health strategic vision across a large mental health trust

**DOI:** 10.1192/bjo.2021.832

**Published:** 2021-06-18

**Authors:** Bethany Cole, Nwaorima Kamalu, Kyra Neubauer

**Affiliations:** Avon and Wiltshire Mental Health Partnership NHS Trust

## Abstract

**Aims:**

Statistically, suicide is less than half as deadly as poor physical health for people with severe mental illnesses (SMI). For every 1000 SMI patients, diseases such as diabetes cause 10-20,000 ‘years of life lost’ compared to 4,000 ‘years of life lost’ to suicide. National charity Rethink dubbed the failure of the NHS to act on this as tantamount to “lethal discrimination”.

We aim to reform the physical health care provision for service users under the care of Avon and Wiltshire Mental Health Partnership NHS Trust (AWP).

**Method:**

To evaluate the current service within AWP, we combined data from a comprehensive audit of 106 inpatients, local Quality Improvement (QI) Projects, and qualitative feedback from a pilot Medical-Psychiatric Liaison Service (MPLS).

**Result:**

Key findings included:

- High rates of physical comorbidities among psychiatric inpatients of all ages
- Novel illnesses occurring during admissions
- Evidence that patients are not receiving adequate physical healthcare from wider NHS
- Junior doctors receiving inadequate support from Seniors and acute Hospital services when managing physical illnesses

Poor recording of cardiometabolic monitoring with few interventions delivered (even when indicated) and challenges finding relevant data in records.

During the MPLS pilot, a Consultant Physician provided virtual ward rounds and advisory sessions. 100% of staff involved reported the service was beneficial for their clinical practice and patient outcomes.

**Conclusion:**

Taking these findings and input from colleagues within AWP and nationally, we created a comprehensive strategic overview on how AWP can deliver high quality physical health care, detailing improvements to make across 5 key domains: Inpatient, Community, Workforce, Education and Information Technology (IT).

Presently, we are working with Clinical Commissioning Groups developing protocols clarifying roles and responsibilities across primary and secondary providers. We are standardising communication between AWP and primary care and expanding links with specialist secondary services (e.g. endocrinology and cardiology). We formed the BRIGHT (Better Recording of Information for Governance and Healthcare in the Trust) project workgroup alongside IT to build safer and more effective records systems.

Medium term recommendations include employing a full-time MPLS Consultant Physician, in addition to ‘Physical Health and Wellbeing Workers’ in all localities, Advanced Nurse Practitioners (working within structured physical care systems) and more allied health professionals (dieticians, speech therapists and physiotherapists).

In the long term, the new Physical Health, IT and QI working groups will maintain development of these proposals, improve training and supervision for clinicians, and achieve healthcare parity for patients across localities.

